# Control of Competence for DNA Transformation in *Streptococcus suis* by Genetically Transferable Pherotypes

**DOI:** 10.1371/journal.pone.0099394

**Published:** 2014-06-26

**Authors:** Edoardo Zaccaria, Peter van Baarlen, Astrid de Greeff, Donald A. Morrison, Hilde Smith, Jerry M. Wells

**Affiliations:** 1 Host-Microbe Interactomics, Animal Sciences, Wageningen University, Wageningen, The Netherlands; 2 Central Veterinary Institute, Animal Sciences, Wageningen University, Lelystad, The Netherlands; 3 Biological Sciences, University of Illinois at Chicago, Chicago, Illinois, United States of America; Centers for Disease Control & Prevention, United States of America

## Abstract

Here we show that *S. suis*, a major bacterial pathogen of pigs and emerging pathogen in humans responds to a peptide pheromone by developing competence for DNA transformation. This species does not fall within any of the phylogenetic clusters of streptococci previously shown to regulate competence via peptide pheromones suggesting that more species of streptococci may be naturally competent. Induction of competence was dependent on ComX, a sigma factor that controls the streptococcal late competence regulon, extracellular addition of a *comX*-inducing peptide (XIP), and ComR, a regulator of *comX*. XIP was identified as an N-terminally truncated variant of ComS. Different *comS* alleles are present among strains of *S. suis*. These *comS* alleles are not functionally equivalent and appear to operate in conjuction with a cognate ComR to regulate *comX* through a conserved *comR*-box promoter. We demonstrate that these ‘pherotypes’ can be genetically transferred between strains, suggesting that similar approaches might be used to control competence induction in other lactic acid bacteria that lack ComR/ComS homologues but possess *comX* and the late competence regulon. The approaches described in this paper to identify and optimize peptide-induced competence may also assist other researchers wishing to identify natural competence in other bacteria. Harnessing natural competence is expected to accelerate genetic research on this and other important streptococcal pathogens and to allow high-throughput mutation approaches to be implemented, opening up new avenues for research**.**

## Introduction

In recent years the acceleration in sequencing of bacterial genomes has made genome sequences available for multiple strains in many species. Comparative analysis of genomes has revealed an important role of horizontal gene transfer (HGT) in the evolution of bacterial genomes [1], [2], [3]. The transfer of genetic information between strains and even species of bacteria can give rise to quantum evolutionary leaps that increase bacterial fitness and their capacity to colonize new ecological niches. Most bacteria appear to be able to transfer genetic information via mobile genetic elements such as bacteriophages, plasmids, or transposons, while to date only about 82 bacterial species have been documented to be naturally transformable [3], [4]. Nonetheless, natural competence is conserved in members of at least six different bacterial phyla suggesting that it is very old in evolutionary terms [4], [5]. This includes the genus *Streptococcus*, one of the most abundant genera in the human small intestine, oral cavity and pharynx [6], [7]. In the gut HGT and natural competence are likely to have played a major role in the evolution and ecology of the microbiota as well as strain diversity [8]. The competent state of “DNA receptivity” is controlled by a large, dispersed set of genes encoding proteins responsible for DNA uptake and recombination, as well as other processes related to competence induced cell stress [9], [10], [11], [12], [13], [14]. Homologues of competence genes are widespread among bacteria that have not yet been demonstrated to be naturally transformable, suggesting that the mechanism may in fact be more common, especially within the phyla that contain members that are already known to be naturally competent. In some bacteria, the competence genes may have become non-functional or evolved such that they are now involved in different processes. An alternative explanation for the presence of competence genes in apparently non-competent bacteria is that they are indeed transformable but only when the right growth conditions and/or specific environmental cues are present. *S. thermophilus* possesses functional homologues of competence genes found in *Streptococcus pneumoniae,* with the exception of *comCDE*, encoding a two-component regulator and competence pheromone of the pneumococcal competence regulon [14]. However, spontaneous competence development in this species was only recently observed, during early exponential phase growth in a synthetic medium, suggesting that competence in this species is regulated by an alternative mechanism to *comCDE* [15]. Indeed, a novel oligopeptide competence pheromone (XIP) characterized by a double-tryptophan motif near the C-terminus [16], as well as its intracellular target protein (ComR) were subsequently identified in this species. The current model predicts that the oligopeptide pheromone is presumably internalized by an Opp transporter enabling it to bind ComR, thereby altering the affinity of this transcription regulator for an inverted repeat motif upstream of the *comX*, encoding an alternative sigma factor. Binding of ComR to the *comX* promoter is then predicted to initiate expression of the ComX sigma factor that associates with the RNA polymerase core and binds to the promoters regulating the late competence genes. Mashburn-Warren et al. [16] demonstrated that a system closely resembling the one operating in *S. thermophilus* is present in *S. mutans* and that homologues of *comS, comR* and *comX* are present in streptococcal species of the pyogenic and bovis groups [17].

In the streptococcus phylogenetic tree, 36 species are grouped into six main clusters with the exception of *S. suis* and *S. acidominimus* [18]. *S. suis*, like its more distant relatives in the genera *Lactococcus* and *Lactobacillus*, possesses homologues of all the streptococcal competence genes involved in DNA uptake and recombination including *comX*, the master regulator of competence [4], [19]. Yet, natural competence development has not been demonstrated in *S. suis* despite a growing body of research on this important zoonotic pathogen [20]. To investigate the possibility that a natural competence system exists within *S. suis* we mined its genome for sequence motifs conserved in the *comX* promoter regions. Here we report results of genomic analysis of predicted promoter sequence patterns of *comX*, and identification of genes encoding an Rgg-like transcriptional regulator ComR, and a cognate pheromone, ComS, in *S. suis*. Moreover, we identified conditions suitable for efficient pheromone activation of natural competence in 5 different serotypes and around 60% of *S. suis* isolates that were otherwise poorly or not at all transformable. This discovery will open up new avenues for genetic analysis of this important pathogen and should assist in discovery of natural competence systems in other bacteria.

## Results

### Identification of an alternative sigma factor gene (comX) and a conserved comX promoter in *S. suis*

**Figure 1:**
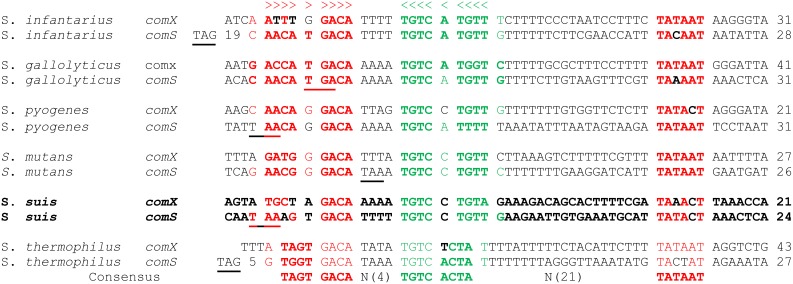
Comparison of *S. suis comS and comX* (*sigX*) promoter regions with those of bovis, pyogenic, mutans, and salivarius groups. The Stop triplet of ComR is underlined. Green and red formatted sequences in the comR-box highlight RNA base-pairing in a potential stem-loop structure. The conserved Pribnow box (TATAAT) at −10 is highlighted in red. Note that the core 12 bases of the ComR box are shared by all species. The distance to the start codon of *comS* or *comX* is indicated by the numbers at the far right.

Inspection of the *S. suis* strain P1/7 genome revealed a single homologue of *comX* (SSU0016) (Table S1) with highest similarity to *comX* of *S. pneumoniae* (46.36%) and *comX* in *S. mutans* (43.23%), both of which mediate expression of the late competence regulon [21]. The *S. suis comX* locus bears strong similarity to the *comX* loci in *S. sanguinis*, *S. pneumoniae*, and *S. gordonii,* with conservation of upstream genes encoding MesJ, hypoxanthine-guanine phosphoribosyltransferase (*Hpt*) and the cell division protease FtsH, and of downstream genes encoding 16 S ribosomal RNA (Fig. S1). In contrast, sequence analysis of the *comX* promoter in *S. suis* revealed a high conservation with *comX* promoters in the bovis and pyogenic groups (Fig. 1). A predicted Pribnow box (−10 element) of *S. suis comX* lies 31 nt upstream of the initiation codon but, as with the other *comX* promoters, the canonical −35 hexamer is missing. The direct-repeat binding site of the competence regulator *comE* is also missing, replaced by the sequence TGTC C/A TGT T/A, characteristic of the ComR box binding site in the mutans, bovis, and pyogenic groups [22], [23]. In *S. suis* the consensus ComR box at the *comX* promoter differs in the most distal inverted repeat sequence upstream of *comX,* which in the bovis and pyogenic streptococcal species forms part of the ‘stem’ of the transcription terminator for an upstream tRNA gene.

### Identification of a comR/comS locus in *S. suis*

**Figure 2:**
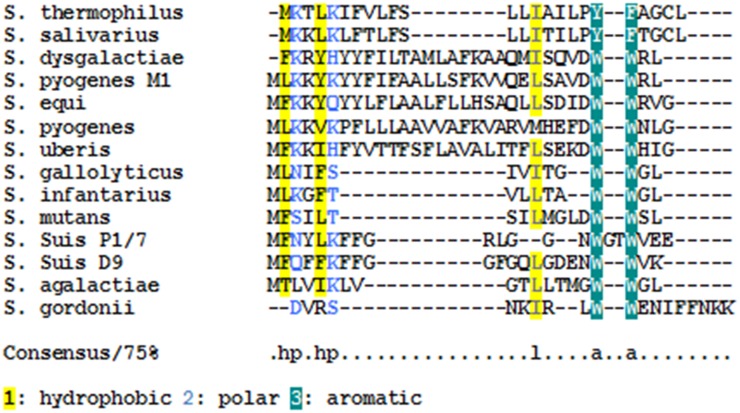
Protein sequences and chemical properties of the known or putative competence pheromones.

As the ComR box (Fig. 1) is instrumental to the regulation of early competence genes *comX* and *comS* in *S. mutans* [16] and *S. thermophilus* (14), we searched the *S. suis* genome for ComR box-containing promoters, using the consensus sequence AAAG N(1, 4) GACA N(4) TGTCCTG N(20) TA N(3) in the Fuzznuc program in the EMBO Open Software suite [24]. This pattern was found in a 405-nt intergenic region between a putative transcriptional regulator (SSU0049) and a transposase gene (SSU0051), in an organization resembling the *comRS* locus of the early competence regulatory systems in *S. pyogenes* and *S. mutans* [16], [17]. This led us to search for *comS*-like open-reading frames downstream of the apparent ComR box. We identified a 63-nt ORF predicted to encode a 21-residue peptide (SSU0050) that was not annotated in GenBank. The conserved genomic context of *comR* and *comS* in *S. mutans* and *S. suis* suggested that SSU0050 could be a *comS* gene. Furthermore the putative *comS* ORF in *S. suis* shared similar amino acid sequence properties to the *S. mutans* ComS [16] (Fig. 2). All *comS* products have a net positive charge (except in the case of *Streptococcus gallolyticus*) and all the competence inducing peptides (XIPs) identified in bovis and pyogenic streptococci and *S. mutans* contain a double-tryptophan (WW) motif near the C-terminus (Fig. 2). The C-terminus of the predicted ComS in *S. suis* however, contains two tryptophan residues separated by 2 other amino acids: W-G-T-W. The ComS sequence is identical in genomes of six of the seven *S. suis* serotype 2 strains known to date. In the serotype 7 strain D9, *comX*, *comR* and the *comX*-like promoters are conserved, but the putative competence pheromone contains a double-tryptophan at the C terminus, as in the pyogenic, mutans, and bovis groups.

### Competence development in *S. suis* is induced by extracellular ComS derivatives and depends on ComR and ComX

**Figure 3:**
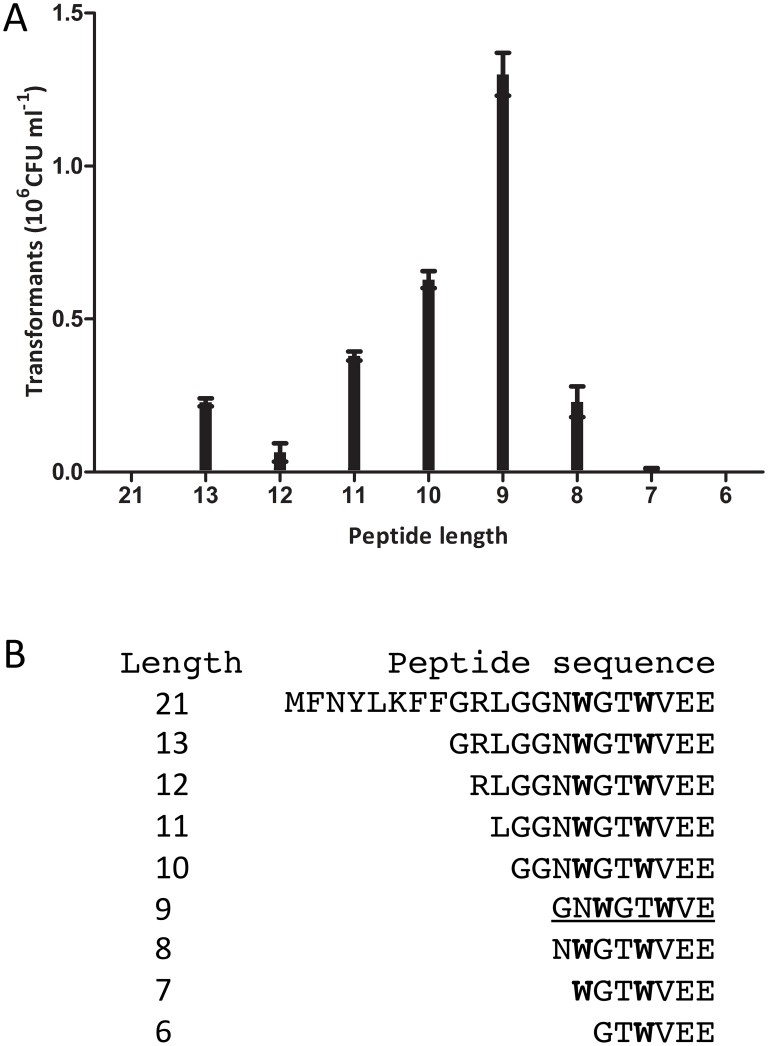
Truncated variants of the predicted ComS peptide and their effect on transformation efficiency. A) Induction of competence by the truncated variants (from 13 to 6 amino acids) of the predicted ComS at 250 µM peptide and 10 ng/µl of the plasmid pNZ8048. Error bars show the standard deviation of 4 different experiments. B) Sequences of full length and truncated variants of the competence inducing peptide.

To test whether *S*. *suis* strain S10 possesses a ComRS-regulated competence system, we added a synthetic 21-amino acid (aa) peptide based on the predicted open reading frame of *comS* (SSU0050) to low density logarithmic phase cultures of *S. suis* in THB for a 2-hour incubation with 10 ng/ul of the broad host range plasmid pNZ8048. We also tested N-terminal truncated variants of the predicted ComS, as many streptococcal peptide pheromones undergo N-terminal processing to generate the active form of the peptide [25], [26], [27], [28]. As shown in Fig. 3A and B, competence for transformation was maximal with ComS13-21 (GNWGTWVEE), suggesting that ComS13-21 is, or closely resembles, the active form of this pheromone. Neither full-length ComS nor the ComS16-21 variant gave rise to transformants. Transformation was abolished in a *comR* KO mutant under all conditions tested, supporting the hypothesis that the competence-inducing properties of ComS peptides depend on ComR. Furthermore, no transformants were obtained using the same procedure with a mutant deficient in *comX,* which in other naturally transformable streptococci regulates expression of genes involved in DNA uptake and recombination [21]. Together, these experiments demonstrate that the 9-amino-acid ComS13-21 stimulated development of a high level of competence in *S. suis* and that the *S. suis* homologues of ComR and ComX are necessary for transformation.

**Figure 4:**
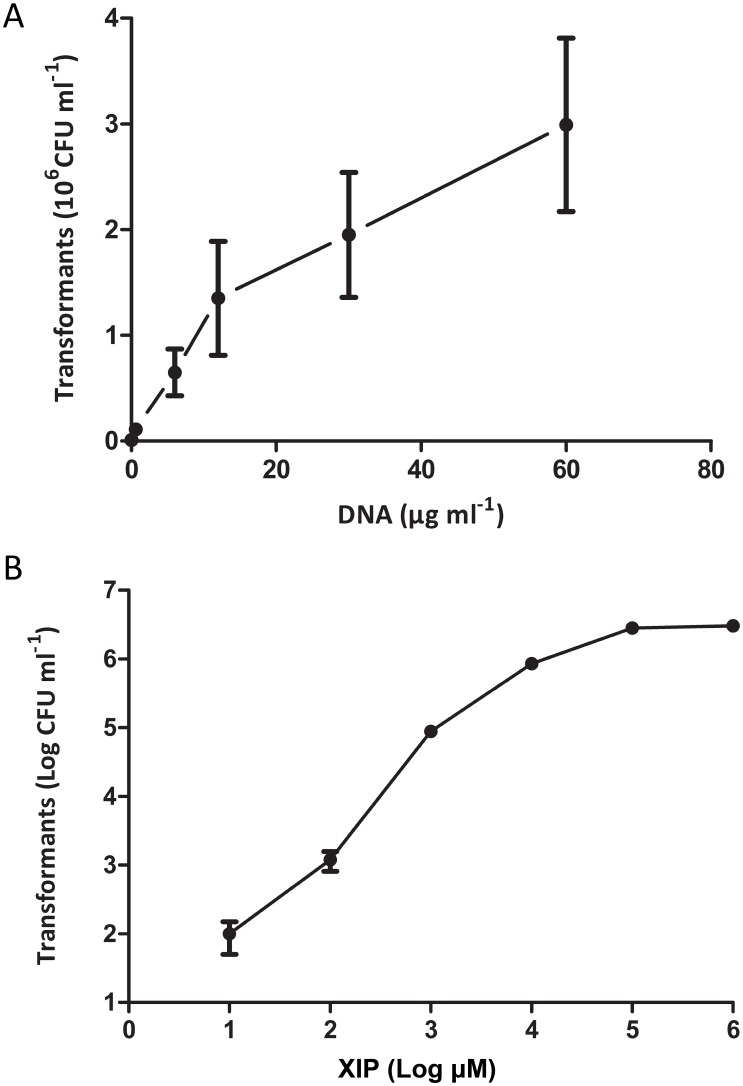
Effect of DNA concentration (A) and competence pheromone concentration (B) on competence development. The experiments were performed using Com13-21 and low-density logarithmic cultures. Error bars show the standard deviation of 4 different experiments.

The effects of varying concentrations of peptide and transforming DNA were investigated using ComS13-21 and low-density logarithmic cultures. Transformation efficiency increased with DNA concentration, giving 1.4 million CFU transformants per ml with 10 µg DNA of pNZ8048, a σ replication mode plasmid [29] (Fig. 4A). The number of transformants also increased with peptide concentration, approaching saturation above 0.2 mM peptide (Fig. 4B). The optimal peptide concentration was higher than previously described for other streptococci [17], [30]. Possible factors affecting efficiency of peptide-induced competence have been documented in previously [31].

### The susceptibility to peptide pheromone is transient and optimal at low bacterial densities

**Figure 5:**
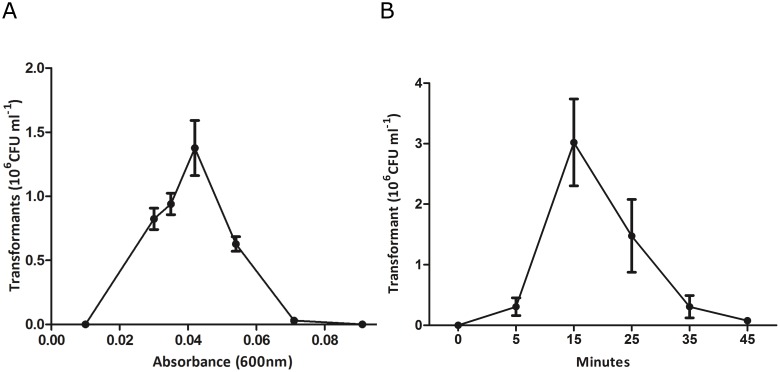
The susceptibility to peptide pheromone is transient and optimal at low bacterial densities. A). Effect of bacterial optical density (O.D. 600 nm) on competence development using a concentration of 10 µg/ml of plasmid DNA (pNZ8048). Competence was not induced over a range of OD values up to 0.3, suggesting that waves of competence do not occur. B). Kinetics of competence induction with plasmid DNA added at the indicated times after ComS13-21 followed 5 min later by addition of DNase (100 U/ml) to degrade any remaining extracellular donor DNA. Error bars show the standard deviation of 4 different experiments.

In other naturally transformable bacteria the state of competence may be transient, depending on the growth conditions and bacterial density in logarithmic growth phase. Commonly, however, this density dependence largely disappears when synthetic peptide is used to obviate the cell-to-cell communication. We determined the transformation efficiency in the presence of the synthetic peptide at different optical densities (O.D. 600 nm) between 0.01 and 0.30 (Fig. 5a) during outgrowth of an overnight culture inoculum. Competence development occurred only within a narrow window of bacterial densities (O.D. 0.03 to 0.06), with maximal transformation efficiency at OD 0.042 (Fig. 5a). To understand better the kinetics of competence development, we determined transformation frequency of bacteria (OD 0.04) at different time-points after addition of ComS13-21 by using 5-min exposure to donor DNA followed by DNase treatment (100 U/ml). The transformation efficiency was low after 5 min incubation with peptide, maximal after 15 min, and then declined to zero by 45 min, suggesting that feedback mechanisms and/or changes in the bacterial density lead to a rapid loss of the capacity for DNA uptake or recombination (Fig. 5b). The large yield of transformants obtained after such brief exposure to DNA indicates that *S. suis* possesses the capacity for a high level of natural competence, with an efficient DNA uptake mechanism.

### Exogenous linear and plasmid DNA transform *S. suis* in the competence state

**Figure 6:**
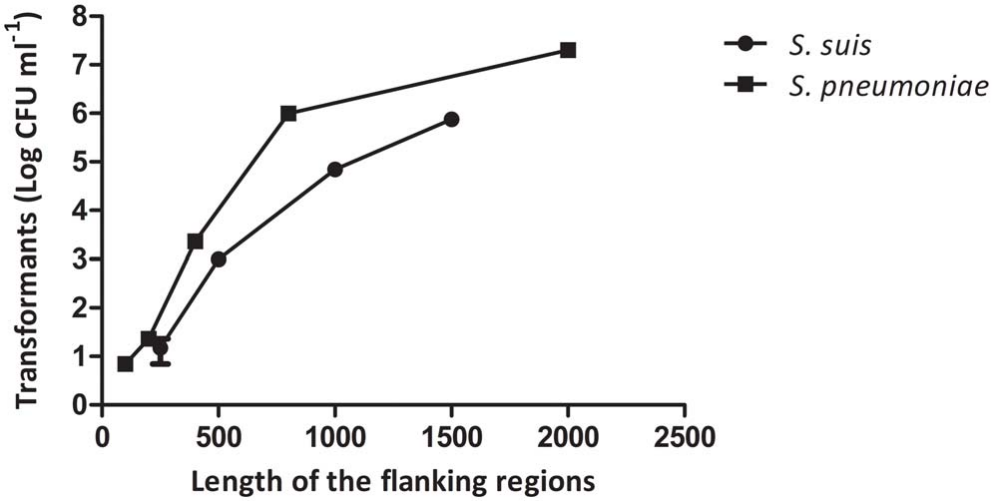
Influence of the length of the homologous flanking DNA on transformation efficiency with linear DNA. The *S. pneumoniae* data was obtained from Lau, at al 2001 [33]. Error bars show the standard deviation of 4 different experiments.

Having established conditions that favor natural competence of *S. suis* by optimizing a transformation protocol using plasmid DNA, we next investigated the efficiency of transformation with linear DNA. For this purpose, we used linear PCR-amplified DNA fragments containing a spectinomycin resistance cassette flanked by sequences identical to 5′ and 3′ coding regions of the *S. suis apuA* gene [32]. To investigate the influence of the length of the homologous flanking DNA on transformation efficiency, the homologous flanking DNA region was varied in length from 250 to 1500 nucleotides. *S. suis* was transformed with the purified PCR products at the concentration of 10 µg/ml in triplicate experiments (Fig. 6). The strong positive influence of the length of the flanking homologous segments on integration efficiency, similar to that reported previously for S. *pneumoniae* [33], suggests that the mechanism of recombination resembles that in *S. pneumoniae*, and provides a practical guide for design of gene-replacement donors in *S. suis*.

### Early competence regulation is pherotype-specific and genetically transferable

We tested the possibility of inducing competence for DNA transformation in different strains and serotypes of *S. suis* using the ComS (aa13-21) pheromone from strain 10. The ability for competence induction was not serotype specific and 10 of the 15 strains could be transformed by the peptide pheromone of strain S10 (Table S2). The genomes of strain S10 and one of the untransformable serotype 7 strains (isolate 7 in Table S2) revealed differences in the sequence of *comS* and *comR*, suggesting that competence is regulated by different peptide pheromones (or XIPs) in some strains that we refer to here as ‘pherotypes’. The coding sequence of the two ComR DNA-binding domains from serotype 2 and serotype 7 strains were identical; all the sequence variation was present in the C-terminal half of the gene, which is hypothesized to interact with XIP. DNA transformation was not induced in serotype 2 strain using the XIP from serotype 7 but became possible after allelic replacement with the serotype 7 *comR/comS* (Fig. S2). As a consequence of this allelic exchange the serotype 2 strain also lost its ability to become competent for DNA transformation with serotype 2 XIP (Fig. S2). These results demonstrate that the *comR/comS* early competence switch can be used to genetically transfer pheromone-specific induction of competence to heterologous strains possessing *comX* coupled to a canonical *comR* promoter, *comX* and the downstream genes necessary for natural competence.

## Discussion

**Figure 7:**
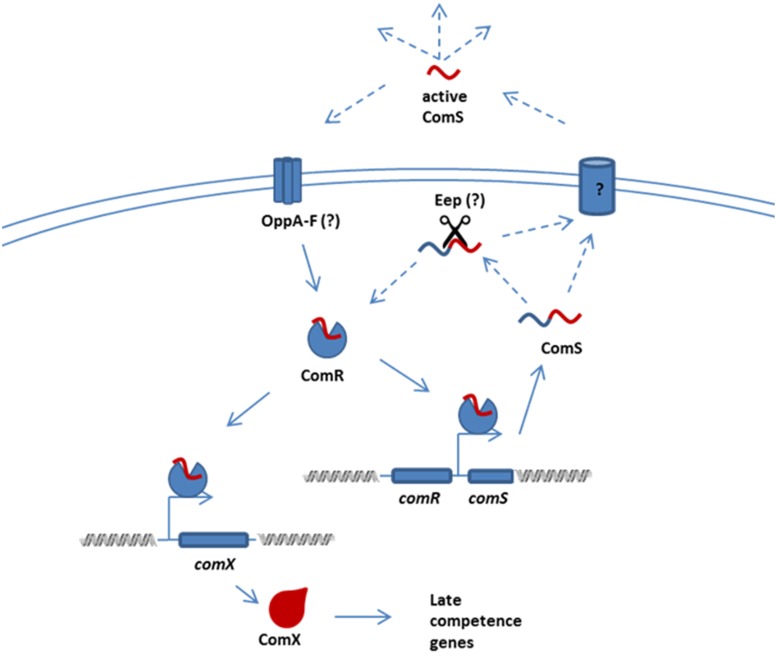
Hypothetical model of competence induction. Given appropriate growth conditions, the transcription of *comS* is activated and the propeptide is produced. ComS is processed before or during transport and its mature form accumulates in the extracellular environment. Uptake of the competence inducing peptide is mediated via an Opp transporter. Intracellular competence inducing peptide activates ComR by binding to its peptide-binding domain leading to transcription of *comS* and of *comX,* the master regulator of the late competence genes involved in the uptake, processing and integration of the extracellular DNA. This hypothetical model of competence induction in S. suis based on published data and data presented in this paper.

Representative species of the mitis, salivarius, mutans, pyogenic, and bovis phylogenetic clusters of streptococci have all been shown to control activity of ComX, a master regulator of bacterial competence, via small peptide pheromones [16], [30], [34], [35], [36], [37]. Here we show that *S. suis*, a streptococcal species which does not appear to fall within any of these phylogenetic clusters, also responds to a peptide pheromone by developing competence for DNA transformation. This finding does suggest that additional streptococcal species might also regulate competence via peptide pheromones (Fig. 7). The competence system in *S. suis* was discovered by searching the genome for the conserved promoter elements found upstream of *comX* and *comS* in *S. thermophilus* and *S. mutans* [16], [17]. Two such promoter regions were identified in the *S. suis* genome (Fig. 1). Downstream of one promoter we identified a homologue of *comX*, the alternative sigma factor that plays a fundamental role in the competence system in *S. thermophilus* and *S. mutans*. Downstream of a second promoter was a small ORF encoding a potential competence pheromone propeptide that we later designated ComS to highlight its homology with the ComS of *S. thermophilus* and *S. mutans*. In the bovis, pyogenes and mutans species of streptococci the competence propeptide sequence is highly variable except for the presence of two adjacent tryptophan residues near the C terminus which appear to be essential for competence induction in *S. mutans* [16] (Fig. 3B). In *S. suis* serotype 2 there are also two tryptophan residues near the C-terminus of the competence peptide but they are separated by two different amino acid residues. As the full length 21-aa ComS propeptide of *S. suis* did not induce competence for DNA transformation under our standard conditions, we hypothesized that the propeptide needed to be processed into a biologically active form, and thus tested several N- terminal deletion variants. Competence induction was optimal using the C-terminal 9 residues of ComS, suggesting that the propeptide contains specific N-terminal sequences that determine processing and secretion steps.

In *S. mutans*, ComS is processed to form a 7-aa comX inducing peptide (13), which has been detected extracellularly in chemically defined medium. Poor activity of the propeptide ComS itself indicates that it is processed before or during transport across the cytoplasmic membrane (29), via unknown mechanisms. It is likely that a similar mechanism occurs in *S. suis* because truncated variants of ComS but not the full length peptide induce competence for transformation when added exogenously. Moreover *S. suis* contains a predicted orthologue of *S. thermophilus* Eep, data not shown, which is involved in processing of the competence peptide [31]. The putative ComS propeptides of *S. pyogenes*, *S. uberis*, *S. dysgalactiae* and *S. equi* possess a basic N-terminus and hydrophobic central core, which are characteristic of type 2 signal secretion leaders, although the polar C-terminus is absent [38]. In contrast the ComS propeptides encoded by *S. suis* and *S. mutans* are hydrophobic shorter versions of those found in streptococci in the pyogenic and bovis groups (17–22 residues) and do not contain N- or C-terminal signatures associated with signal secretion leaders. The existence of propeptides with different chemical properties suggests that these propeptides might be secreted and processed by different mechanisms.

The competence-inducing peptides of *S*. *mutans* and *S*. *thermophilus* are imported into the cytoplasm by the ATP-dependent multi-subunit transporters known as Opp or Ami [15], [16], [22], [39]. As the competence-inducing peptide of *S. suis* functions when added exogenously, we predict that the homologues of the *S. suis* Opp multi-subunit transporter (Table S1) are also involved in import of XIP into the cytoplasm for access to ComR.

This study builds on the discovery of a pheromone-regulated natural competence system in *S. mutans* [16] and *S. thermophilus* [17], [31] and exemplifies the approaches that can be taken to identify competence regulatory circuits in bacteria possessing homologues of *comX*. Many streptococcal competence systems similar to those of *S. mutans* and *S. thermophilus* possess a conserved ComR box promoter upstream of both *comX* and *comS*. The cognate *comS* ORFs are frequently not annotated due to their small size and can be difficult to identify in bacterial genomes. However homologues of *comX* are commonly annotated when present and, as we show here, genome homology searches with the promoter regions upstream of *comX* can help to identify ORFs encoding ComS peptides. As N-terminal truncations may be necessary for ComS to induce competence, a series of truncated variants of ComS should be tested at concentrations up to 250 µM. Roadblocks may well remain in determining the optimal environment for competence development. For example, the response to XIP requires expression of peptide transporters and of *comR*, as well as freedom from interfering activities such as proteases or nucleases. Therefore ComS peptides should be tested with bacteria grown in a variety of culture media and in multiple phases of growth [40].

ComR has been shown to be necessary for ComS to induce competence and on the basis of its sequence similarity to the PlcR transcriptional regulator we assume that it also forms a dimer or tetramer by directly binding ComS13-21, to form an active transcriptional activator. In *S. suis*, *S. mutans* and *S. thermophilus* ComR is annotated as an Rgg–like transcriptional regulator of which there may be several paralogues in a bacterial genome [36], [37]. In the *comRS* regulatory circuits identified to date *comR* was upstream of *comS* but in other organisms it may be at a distant locus. It is also possible that *comR* itself may be regulated by environmental factors highlighting again the need to test for competence induction using a range of growth conditions including multiple carbon sources. An alternative strategy is to increase copy number of *comR* by transfer to a plasmid, which in *S. mutans* increased transformability in the presence of *comS* [16].

The existence of different pherotypes divides competent *S. suis* into bacterial populations that allow pheromone communication to take place only within, not between, populations. Nevertheless, pherotype switching could occur by uptake of DNA and gene replacement within the species or even between species that utilize the comS, comR regulatory switch. Thus pherotype switching may be a biologically important mechanism, that enables different species to be induced to competence by the same XIP. Our finding that genetic transfer of *comS/comR* from an XIP-responsive strain to a non-responsive strain confers XIP-specific induction of competence suggests an approach to regulate competence development in the species of lactic acid bacteria that appear to possess homologues of *comX* and the late competence genes found in naturally competent streptococci but no homologues of the *comR/comS* early competence switch (Table S1). This would require coupling of the canonical *comR* promoter to the *comX* of the host species and introduction of *comR/comS* from bacteria with demonstrated natural competence such as *S. suis*. Nevertheless, additional factors may be required for activation as previously shown for *S. pneumoniae* where ComW is required for stabilization and activation of the alternative sigma factor ComX [41].

The current low-efficiency genetic approach to manipulate *S. suis* requires use of *E. coli* shuttle vectors and suicide vectors. Thus there was an urgent need for novel methodology that would allow more rapid genetic manipulation in this important zoonotic pathogen, which is the major cause of bacterial meningitis in adults in Vietnam [42]. *S. suis* provokes more than 300 million dollars of economic losses in the USA pork industry alone (33). The competence system identified in this paper allows high frequency of transformation of *S. suis* and the possibility to use linear DNA fragments assembled using common PCR-based approaches for rapid targeted gene modification. This will overcome existing problems with low transformation efficiency. Routine genetic manipulation and gene deletion in *S. suis* would allow high throughput mutation approaches to be implemented, opening up new avenues for research on this important pathogen.

## Materials and Methods

### Bacterial strains, plasmid and growth conditions

The bacterial strains and plasmids used in the present study are listed in Table S3. *S. suis* strains were grown in Todd-Hewitt broth (THB) (Difco) at 37°C under 5% CO_2_. When required, chloramphenicol (5 **µ**g/ml) or spectinomycin (100 **µ**g/ml) was added to the media. Solid agar plates were prepared by adding 12 g/L agar to the medium.

### Genome analysis

BLAST searches with *S. suis* genome sequences were performed using non-redundant sequences accessible at the National Centre for Biotechnology Information internet site (http://www.ncbi.nlm.nih.gov). Specific promoter patterns in nucleotide sequences were identified using the Fuzznuc program in the EMBO Open Software suite [24]. Comparative genome analysis among *S. suis* strains was performed using MicrobesOnline (http://www.microbesonline.org) [43]. Sequence alignments were made using ClustalW (http://www.ebi.ac.uk/Tools/clustalw2/index.html) [44]. Peptide alignments were annotated using CHROMA [45].

### Preparation of synthetic peptides

Peptides were purchased from JPT Peptide Technologies (Berlin, Germany) at purity grades of 62–89%. Transformation efficiencies might have been higher with highly pure peptides as the preparations used in this study were between 62 to 89% pure. Stock solutions were dissolved in Milli-Q water at a final concentration of 5 mM, taking in consideration their specific purity. Stock solutions were stored in 50-µl aliquots at −80°C.

### Natural transformation experiments

*S. suis* strains were grown overnight in THB broth at 37°C under 5% CO_2_. The overnight culture was diluted 1∶40 into similar pre-warmed media, and grown at 37°C without shaking. 100-**µ**l samples were removed from the main culture after 1 hour (with an O.D. at 600 nm between 0.035 and 0.058).

Donor DNA (1.2 **µ**g of pNZ8048) in EB buffer (10 mM Tris-Cl, pH 8.5) was added to the bacteria along with 5 **µ**l of stock peptide at a final concentration of 250 **µ**M. After 2 hours of incubation at 37°C under 5% CO_2_ in 1.5 ml Eppendorf Safe Lock Tubes™, the samples were diluted and plated in THB agar plates with the required antibiotics.

For the study of the kinetics of competence induction, plasmid DNA was added at the indicated times after ComS13-21 followed 5 min later by addition of DNase at a concentration of 100 U/ml (Qiagen Ltd,. Crawley, UK) to limit exposure to transforming DNA to 5 min.

### Insertional inactivation of *apuA*

Primers used for mutagenesis are listed in Table S3. The plasmid pG9-*apuA : : spc* [*32*] *was used as a template for PCR to obtain a linear DNA fragment carrying the apuA gene interrupted by the spectinomycin cassette. 1 µg of the purified fragment was used to transform S. suis strain 10 with selection on* agar containing 100 µg/ml of spectinomycin at 37°C; the double crossover events were verified by PCR using the primer pairs CtrlMutA1/CtrlR1 and CrtlMutB1/CtrlR2.

To study the effect of length of the homologous arms to the transformation efficiency four different PCR fragments with increasing length were synthesized using the primer pairs described in Table S3.

### Construction of mutant strains

Mutants in *comX* and *comR* were constructed by transformation of *S. suis* S10 with linear DNA fragments comprising the spectinomycin resistance flanked by about 1 kb of DNA with homology to sequences adjacent to the target gene. The linear DNA fragments were generated by SOE-PCR using the primers listed in Table S4.

## Supporting Information

Figure S1 Schematic view of *ComX* and *comRS* regions in *S. suis* and other streptococcal species. A) Overview of the *comX* region in the genome of different streptococcal species. The *comX* sequences have a sequence identity greater than 37%. Orthologous genes are represented with the same colors if amino acid identity was greater than 50%. B). Overview of the *comRS* locus in *S. suis* and in other representative streptococcal species. The *comR* sequences have a sequence identity greater 37%. Orthologous genes are represented with the same colors if amino acid identity was greater than 50%. (Red box): *comR* genes, (black boxes): conserved genes surrounding *comR.*
(PDF)

Figure S2 Competence regulation is pherotype-specific and genetically transferable. DNA transformation could be induced in serotype 2 strain S10 using its own XIP2 but not that of strain 7 (serotype 7). After allelic exchange of the comR/comS from strain 7 into strain S10 (S. suis S10 XIP7) high efficiency of transformation was obtained with XIP7 but not XIP2, demonstrating that the strain 7 comR/XIP7 genetic switch (pherotype) for competence induction was functional and transferable. In all the successful transformations the numbers of transformants obtained were of the same order of magnitude.
(DOC)

Table S1 Conservation of competence genes in streptococci, *Lactococcus lactis, Lactobacillus plantarum* and *Bacillus subtilis.*
(PDF)

Table S2 Overview of *S. suis* isolates and their ability for competence for DNA transformation to be induced using the synthetic ComS peptide. *serotype 1 reference strain.
(DOCX)

Table S3 Bacterial strains and plasmids used in this study.
(DOCX)

Table S4 Oligonucleotide primers used in this study.
(DOCX)

File S1 References in supporting files.
(DOCX)
