# Supplementary material for: Control of Competence for DNA Transformation in Streptococcus suis by Genetically Transferable Pherotypes
Source: PLoS One. 2014 Jun 26;9(6):e99394. doi: 10.1371/journal.pone.0099394 (PMC4072589; doi:10.1371/journal.pone.0099394)
Supplement: Figure S1 — Schematic view of ComX and comRS regions in S. suis and other streptococcal species. A) Overview of the comX region in the genome of different streptococcal species. The comX sequences have a sequence identity greater than 37%. Orthologous genes are represented with the same colors if amino acid identity was greater than 50%. B). Overview of the comRS locus in S. suis and in other representative streptococcal species. The comR sequences have a sequence identity greater 37%. Orthologous genes are represented with the same colors if amino acid identity was greater than 50%. (Red box): comR genes, (black boxes): conserved genes surrounding comR. (PDF) [file pone.0099394.s001.pdf]

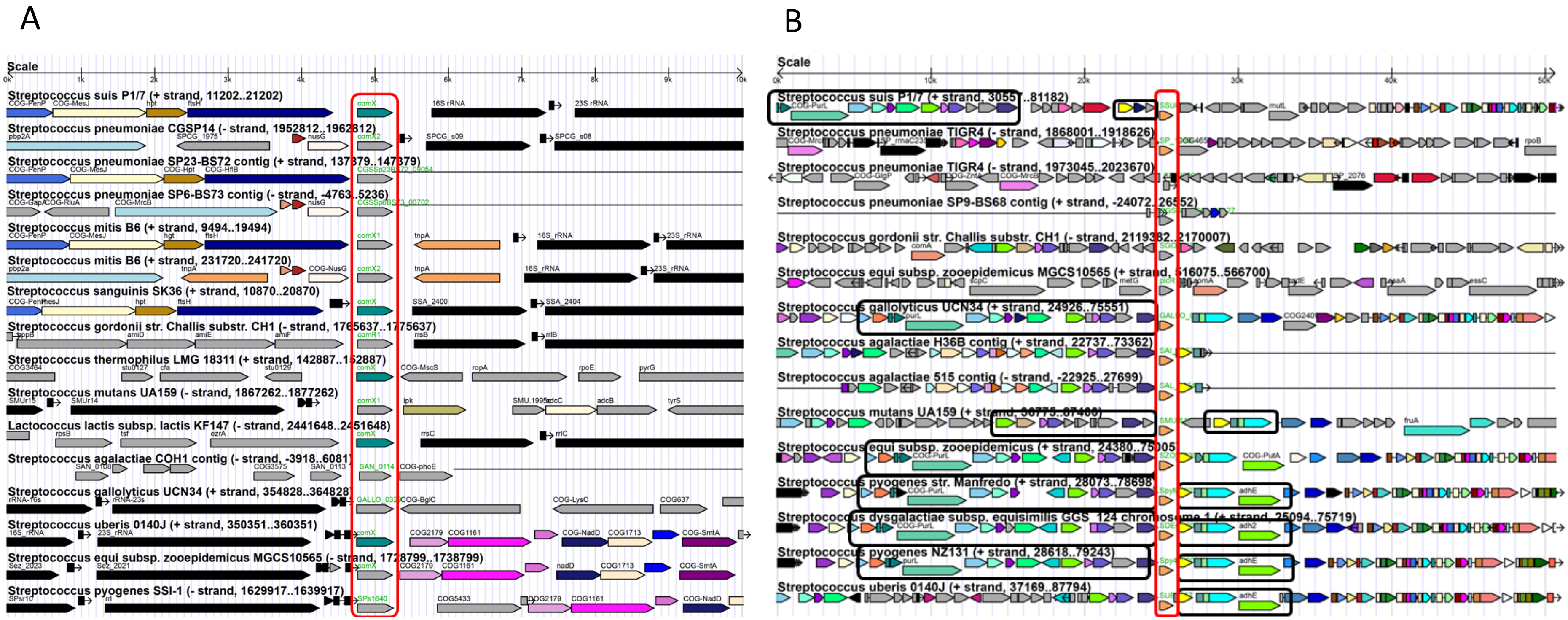

Figure 1. A) Overview of the *comX* region in the genome of different streptococcal species. The *comX* sequences have a sequence identity greater than 37%. Orthologous genes are represented with the same colors if amino acid identity was greater than 50%. B). Overview of the *comRS* locus in *S. suis* and in other representative streptococcal species. The *comR* sequences have a sequence identity greater 37%. Orthologous genes are represented with the same colors if amino acid identity was greater than 50%. (Red box): *comR* genes, (black boxes): conserved genes surrounding *comR*.
