# Supplementary material for: Control of Competence for DNA Transformation in Streptococcus suis by Genetically Transferable Pherotypes
Source: PLoS One. 2014 Jun 26;9(6):e99394. doi: 10.1371/journal.pone.0099394 (PMC4072589; doi:10.1371/journal.pone.0099394)
Supplement: Figure S2 — Competence regulation is pherotype-specific and genetically transferable. DNA transformation could be induced in serotype 2 strain S10 using its own XIP2 but not that of strain 7 (serotype 7). After allelic exchange of the comR/comS from strain 7 into strain S10 (S. suis S10 XIP7) high efficiency of transformation was obtained with XIP7 but not XIP2, demonstrating that the strain 7 comR/XIP7 genetic switch (pherotype) for competence induction was functional and transferable. In all the successful transformations the numbers of transformants obtained were of the same order of magnitude. (DOC) [file pone.0099394.s002.doc]

A.

B.

Figure S2. MALDI TOF Mass Spectra of peptides from bacterial cell pellet (A) and *S. suis* culture supernatant (B and C). In all panels the top mass spectrum shows sample complexity in the range 700 Da to 8000 Da. The inserts show an expanded spectrum from 2470 Da to 2700 Da (panels A and B) and 1000 to 1100 (panel C). The bottom mass spectrum is expanded to show the putative full length ComS (arrowed in panels A and B) and truncated form of ComS inducing competence for transformation (arrowed in panel C). Samples for mass spectrometry were passed through a C18 Solid Phase Extraction (SPE) column and the resulting eluents applied to an analytical Reversed Phase HPLC column running an 25-46% acetonitrile 0.1% TFA gradient over 45 minutes. Fractions were collected at 1 minute intervals and assessed for the presence of peptides of interest using MALDI TOF Mass Spectrometry. Synthetic peptides were used as reference standards.
