# Supplementary material for: Control of Competence for DNA Transformation in Streptococcus suis by Genetically Transferable Pherotypes
Source: PLoS One. 2014 Jun 26;9(6):e99394. doi: 10.1371/journal.pone.0099394 (PMC4072589; doi:10.1371/journal.pone.0099394)
Supplement: Table S1 — Conservation of competence genes in streptococci, Lactococcus lactis, Lactobacillus plantarum and Bacillus subtilis. (PDF) [file pone.0099394.s003.pdf]

**Putative Function**	**Gene in *S. suis***	***S. pneumoniae***	***S. pyogenes***	***S. mutans***	***S. thermophilus***	***L. lactis***	***L. plantarum***	***B. subtilis***
**Transcriptional control**								
First transcriptional regulator	*comR* (SSU0049)	37.46%	35.93%	43.81%	34.43%	-	-	-
Master regulator	*comX* (SSU0016)	46.36%	40.00%	43.23%	40.52%	32.71%	30.86%(*sigH*)*	*comK**
**DNA uptake-recombination machinery**								
Helicase required for DNA uptake	*comFA* (SSU0393)	66.36%	50.00%	57.87%	52.79%	51.20%	38.95%	37.35%
Membrane DNA-binding receptor	*comEA* (SSU0394)	47.95%	47.59%	53.41%	59.26%	37.56%	46.34%	37.56%
Competence protein	*coiA* (SSU1083)	51.60%	45.19%	50.48%	46.01%	42.50%	39.11%	31.91%
Component of the DNA transport	*comGC* (SSU0128)	70.37%	55.68%	60.23%	60.23%	56.82%	43.37%	49.33%
Competence associated endonuclease	*endA* (SSU1009)	69.49%	65.47%	65.38%	61.56%		35.29%	
DNA transporter	*comFC* (SSU0394)	49.08%	40.64%	50.91%	44.09%	45.25%	35.24%	31.28%
**Signalling function**								
Sensing and regulation	------	*ComD-E*	-	-	-	-	-	*comA-comP*
Peptide export	------	*ComA-B*	-	-	-	-	-	*comQ*
**Possible pheromone-processing and peptide transporter**								
Pheromone-processing	*eep* (SSU1754)	63.25%	64.29%	62.29%	65.00%	54.00%	47.73%	37.94%
Oligopeptide binding protein	*oppA* (SSU1664)	-	-	22.69%	-	32.26%	-	24.14%
	------	66.82%	49.92%	-	AmiA1	26.97%	28.43%	-
Oligopeptide transporter								
	oppB(SSU1661)		33.44%	35.74%		52.66%		39.69%
	oppC (SSU1660)	32.51%	31.67%	32.16%	32.39%	51.45%	32.89%	33.78%
	oppD (SSU1663)	45.03%	46.03%	47.63%	44.27%	55.11%	46.84%	48.28%
	oppF(SSU1662)	47.73%	47.53%	47.55%	49.79%	60.13%	47.91%	50.78%

Table S1. Conservation of competence genes in streptococci, *Lactococcus lactis*, *Lactobacillus plantarum* and *Bacillus subtilis*
