# Supplementary material for: Control of Competence for DNA Transformation in Streptococcus suis by Genetically Transferable Pherotypes
Source: PLoS One. 2014 Jun 26;9(6):e99394. doi: 10.1371/journal.pone.0099394 (PMC4072589; doi:10.1371/journal.pone.0099394)
Supplement: Table S2 — Overview of S. suis isolates and their ability for competence for DNA transformation to be induced using the synthetic ComS peptide. *serotype 1 reference strain. (DOCX) [file pone.0099394.s004.docx]

| ***S. suis* isolate** | **Serotype** | **Virulent** | **Competence induced with S10 pherotype** |
| --- | --- | --- | --- |
| 6388 | 1 | ND | yes |
| 6112 | 1 | ND | yes |
| 6555* | 1 | ND | no |
| S10 | 2 | yes | yes |
| P1/7 | 2 | yes | yes |
| 05ZYH33 | 2 | yes | yes |
| 7917 | 7 | ND | yes |
| 15009 | 7 | ND | yes |
| 7711 | 7 | ND | no |
| 8074 | 7 | ND | no |
| 7 | 7 | ND | no |
| 7997 | 9 | ND | yes |
| 8067 | 9 | ND | yes |
| 22083 | 9 | ND | no |
| 13730 | 14 | ND | yes |

Table S2. Overview of *S. suis* isolates and their ability for competence for DNA transformation to be induced using the synthetic ComS peptide. *serotype 1 reference strain.
