# Supplementary material for: Control of Competence for DNA Transformation in Streptococcus suis by Genetically Transferable Pherotypes
Source: PLoS One. 2014 Jun 26;9(6):e99394. doi: 10.1371/journal.pone.0099394 (PMC4072589; doi:10.1371/journal.pone.0099394)
Supplement: Table S3 — Bacterial strains and plasmids used in this study. (DOCX) [file pone.0099394.s005.docx]

**Plasmid or strain Description Reference**

pNZ8048 *E. coli*- *L. lactis* shuttle vector containing PnisA promoter, CmR (chloramphenicol resistant) (1) pG9*-*apuA pGhost+9 derivative containing 3020 bp of *apuA* locus and *spc* (spectinomycin resistant) (2)

*S. suis* S10 Virulent serotype 2 strain S3881 (3)

*S. suis* mut1 Isogenic *apuA::spc* mutant of strain S10 (2)

*S. suis* mut2 Isogenic *comX::spc* mutant of strain S10 This work

*S. suis* mut3 Isogenic *comR::spc* mutant of strain S10 This work

*S. suis* pNZ *S. suis* strain 10 harboring pNZ8048 plasmid This work

*S. suis* 7

*S.* suis S10 (XIP 7)

Serotype 7 strain S8039

Isogenic *comR/S S10::comR/S 7 spc* mutant of strain S10

(4)

This work

**Table S3 Bacterial strains and plasmids used in this study**
