# Supplementary material for: Control of Competence for DNA Transformation in Streptococcus suis by Genetically Transferable Pherotypes
Source: PLoS One. 2014 Jun 26;9(6):e99394. doi: 10.1371/journal.pone.0099394 (PMC4072589; doi:10.1371/journal.pone.0099394)
Supplement: Table S4 — Oligonucleotide primers used in this study. (DOCX) [file pone.0099394.s006.docx]

| **Primers** | **Sequences** | **Restriction sites** | **Location** |
| --- | --- | --- | --- |
| ApuA250F | CTGAGAATACCGCAACCG |  | 1875397-  414 |
| ApuA250R | GAGTGAGTACGCGTCAAG |  | 1875936-  953 |
| ApuA500F | AGGATGGCGGAACTGTTTG |  | 1875047-  065 |
| ApuA500R | ATTGTGGACCCAGCAGAAG |  | 1876207-  225 |
| ApuA1000F | CCAGCACTTGACCCTTAAGC |  | 1874599-  618 |
| ApuA1000R | AGCAACAAGGTGCGAGTCC |  | 1876675-  693 |
| ApuAR | TACCGATGGCAATTATGAT |  | 1877138-  156 |
| ApuAF | CTTGTCCAGACGCTTGAG |  | 1874137-  154 |
| ComXAF | TTCTGAACGAAGCAGCCCTTGTAG |  | 14857-880 |
| ComXBR | CACCAGACTTCAGCGTTCTACTTG |  | 17027-50 |
| ComXEF | TGAGAGCTCCAGGACATTTTTGTCTAGCATAC | SacI | 15889-909 |
| ComXFR | GGCCTCGAGTAGTTACTAACATCACGTTAATCGAAT | XhoI | 16441-467 |
| SpecIAF | TCCGAGCTCCAGCTTGATGCCTGCA | SacI | 3-18 |
| SpecIAR | CGCCTCGAGATCTGATTACCAATTAGAATG | XhoI | 1166-187 |
| CtrlMutA1 | TGGGTGTGATTTTGGATGTG |  | 1875779-  798 |
| CtrlMutB1 | TAAAGGCCAGCTCAATTGCT |  | 1875595-  554 |
| CtrlF1 | GGTTGGTCGTCCGGATGTTAAAGG |  | 14720-743 |
| CtrlR1 | GCGTTACTCACCCGTTCGCAACTC |  | 17067-090 |
| Ctrl2F | GGAGGATGATTCCACGGTACCATTTC |  | 615-631 |
| Ctrl2R | TATTGCGGGAAATGCAGTGG |  | 938-357 |

Table S4. Oligonucleotide primers used in this study.
