# Supplementary material for: Control of Competence for DNA Transformation in Streptococcus suis by Genetically Transferable Pherotypes
Source: PLoS One. 2014 Jun 26;9(6):e99394. doi: 10.1371/journal.pone.0099394 (PMC4072589; doi:10.1371/journal.pone.0099394)
Supplement: File S1 — References in supporting files. (DOCX) [file pone.0099394.s007.docx]

References in Supporting files

1. Goodstadt L & Ponting CP (2001) CHROMA: consensus-based colouring of multiple alignments for publication. Bioinformatics 17(9):845-846.

2. Ferrando ML, Fuentes S, de Greeff A, Smith H, & Wells JM (2010) ApuA, a multifunctional alpha-glucan-degrading enzyme of Streptococcus suis, mediates adhesion to porcine epithelium and mucus. Microbiology 156(Pt 9):2818-2828

3. Vecht U, Arends JP, van der Molen EJ, & van Leengoed LA (1989) Differences in virulence between two strains of Streptococcus suis type II after experimentally induced infection of newborn germ-free pigs. American journal of veterinary research 50(7):1037-1043.

4. Zhang A, et al. (2011) Comparative genomic analysis of Streptococcus suis reveals significant genomic diversity among different serotypes. BMC genomics 12:523.
